# Pain management of hip osteoarthritis with corticosteroids vs injection therapies: a systematic review and meta-analysis

**DOI:** 10.1186/s12891-025-08666-0

**Published:** 2025-05-15

**Authors:** Euan Kelly, Neil Heron

**Affiliations:** Queens University Belfast, Belfast, UK

## Abstract

**Introduction:**

Osteoarthritis (OA) is the most common degenerative joint disease, characterized by chronic pain and articular cartilage damage. Hip OA is characterized by the progressive breakdown of articular cartilage within the hip, particularly the hip joints ball and socket structure, Hip OA leads to joint pain, stiffness and causes functional limitations.

**Aim:**

To analyse the effectiveness of intra-articular corticosteroids (IA CS) use against other injection therapies for the symptomatic management of hip OA.

**Methods:**

PROSPERO registered—CRD42024526221. Medline, Embase, Scopus and Web of Science were searched for trials. Inclusion Criteria: Adults with symptomatic hip OA, randomised trials for treatment of hip OA with IA injection methods. Studies must involve comparators and the outcome measure must include a measurement of pain such as Western Ontario and McMaster Universities Osteoarthritis Index (WOMAC) or the visual analogue scale (VAS). Cochrane risk of bias and JBI critical appraisal tools assessed risk of bias. RevMan was used for creation of statistical figures within the meta-analysis.

**Results:**

Data extracted in the systematic review presented improvements on pain, function, stiffness, and overall scores using WOMAC and VAS scales. However, data from the meta-analysis indicates that there is no statistical significance (significance is a p value < 0.05) between corticosteroids and placebo or hyaluronic acid (HA). Meta-analyses produced *p* values of 0.05 when comparing CS and placebo WOMAC pain scores at 2 months, CS and HA overall WOMAC at 6 months—*p* value of 0.46. WOMAC stiffness and function scores between CS and placebo at 2 months—p value of 0.05 and 0.08, thus statistically insignificant.

**Conclusion:**

This meta-analysis shows that IA corticosteroid injections for hip OA don’t provide statistically significant symptomatic improvement for patients compared to placebo. Showing the urgent need to assess other therapies in hip OA treatment.

## Introduction

### Background

Osteoarthritis (OA) is the most common degenerative joint disease and a leading cause of disability, characterized by chronic pain and articular cartilage damage [1, 2]. OA affected 595 million people globally in 2020, an increase of 132.2% since 1990 [1]. OA is a complicated disease that can affect any joint in the body such as the knee and hip [3], obesity contributes to OA development by increasing stress on joints [4]. Hip OA is characterized by the progressive breakdown of articular cartilage within the hip, particularly the hip joints ball and socket structure, Hip OA leads to joint pain, stiffness and causes functional limitations [1, 3].

OA affects the entire joint with degenerative processes leading to permanent damage of articular cartilage and painful swelling as it develops [2, 5]. Pain and limited mobility lead to a reduction in patient activity levels, causing muscle weakening and atrophy and therefore further deterioration of the joint, which is known as the physical inactivity pain cycle [6]. OA is difficult to treat due to a lack of effective therapies, with available drugs being associated with side effects and toxicities [2, 7]. Due to the prevalence of OA and the lack of disease modifying drugs it is necessary that viable pain management solutions are found. Conventional treatment, following UK national guidelines involves the use of intra articular corticosteroids (IA CS) with local anesthetics and exercise [8].

### Current treatment modalities

The primary treatments provided to delay surgery and manage pain in hip OA is IA CS and exercise [8]. Multiple IA CS injections are available and prescribed such as triamcinolone acetate [9]. CS provide anti-inflammatory and immunosuppressive effects, they act directly on nuclear steroid receptors preventing accumulation of inflammatory cells, phagocytosis, and inflammatory mediator secretion such as prostaglandins [10]. The anti-inflammatory effect reduces swelling, tenderness, heat, and pain leading to increased joint mobility in OA patients [11, 12]. Preventing inflammation leads to reduced vascular permeability which is essential for tissue health [13], inhibits inflammatory cell accumulation and inflammatory mediators [10], thereby minimizing swelling, tenderness, and erythema of the joint [12]. IA CS are considered effective and frequently prescribed to relieve symptoms in OA patients, although this is reported to occur only in the short term, with some authors reporting 6 weeks [14], and the NICE guidelines stating 2–10 weeks [15]. Exercise has been shown to effectively reduce pain and improve physical function having similar effects to analgesics [16]. However, due to pain causing limited mobility many patients are unable to exercise [17]. The American college of Rheumatology subcommittee on OA recommends CS as a treatment for managing pain [18], but a Cochrane review noted a lack of evidence for functional improvements after IA injections of CS [19]. Studies have also demonstrated negative side effects with IA CS causing joint degradation, especially with repeated injections damaging articular cartilage [17] and repeat high doses (> 3 mg) causing further cartilage damage and chondrotoxicity [20].. Due to the side effects of IA CS, alternative treatments options are necessary. Alternative injection therapies could include platelet rich plasma (PRP), Hyaluronic acid (HA), and other biologics for the management of hip OA pain and symptom management [9].

### Rationale for injection therapies

Injection therapies have a massive role within the United Kingdom’s National Health Service (NHS), being a core treatment for short term reduction in joint related conditions such as OA [9, 22]. Figures from the NHS schedule of costs from 2019/20 show the price of anatomically guided injections being as high as £752 and image guided injections costing £826 [23]. Considering over 8.5 million people [24] are affected in the UK by OA and the regular injections required there is a necessity to evaluate other options for treatment of hip OA. It is necessary to compare IA CS to saline and review if pain relief is substantial or only placebo based. A comparison of IA CS to other biologic therapies is required to conclude which therapy is the most effective option for long term pain relief, mobility, and stiffness. Biologic therapies can be defined as substances produced by living organisms that are used for disease diagnosis, prevention, and treatment, including antibodies and interleukins [25].

An important therapy to evaluate is HA. In an osteoarthritic joint, HA is reduced [26]. HA naturally exists in human tissues such as the umbilical cord, epidermis, and synovial fluid, being a natural fluid that lubricates and cushions the joints [26].. In Exogenous HA can improve chondrocyte synthesis of endogenous proteoglycans and HA thus preventing cartilage degradation and stimulating regeneration. Studies have shown HA can reduce the production of proinflammatory mediators, reducing nerve impulses and sensitivity that is associated with pain in OA [27].

Data indicates that IA HA provides pain relief that is similar, or greater than IA CS, physical therapy and exercise [28]. Placebo has been shown to effectively reduce pain through IA saline injections [29]. However, several trials have shown HA to be more effective in providing pain relief and mobility compared to saline [30–34]. Further Studies have also shown HA injections to be safe and absent from systemic effects [35, 36].

Placebo is the injection of an inert substance such as saline, local anesthetic and water injections, the placebo effect is the physiological response that follows the administration of a placebo injection [37]. Many mechanisms contribute to placebo effects such as expectations, motivation, conditioning, and learning [38, 39]. Placebos are essential in clinical trials as they provide a control to the experiment providing a comparison and therefore a researcher can conclude if a drug is effective, and the results aren’t due to placebo. Placebo controlled trials are regarded as the gold standard for testing new treatment efficacy [40].

### Aims

The primary aim is to compare and investigate the effectiveness of IA CS injections against other injection therapies such as HA, saline and placebo injections, for pain management in adults with hip OA. Secondary aims are to identify the injection that is most effective in improving joint mobility and function in hip OA.

## Methodology

### Research design

This review was prospectively registered with PROSPERO—CRD42024526221. The review followed the Preferred Reporting Items for Systematic Reviews and Meta-analyses (PRISMA) guidelines, as seen in Appendix 1/2. A systematic review and meta-analysis were chosen as the most effective method of analysing current available research by increasing the quality of papers to be used through inclusive and exclusive criteria and reducing the risk of bias as much as possible.

### Inclusion and Exclusion Criteria

Inclusion Criteria: Adults with symptomatic hip OA of all ethnicity, age and gender, that have undergone randomised trials for treatment of hip OA with IA injection methods. Studies must’ve been randomised trials that involve comparators, blinding and must be extractable for one measurement of pain such as Western Ontario and McMaster Universities Osteoarthritis Index (WOMAC) or the visual analogue scale (VAS).

Exclusion Criteria: Any studies on animals were excluded. Trials without comparators, randomisation, co-interventions, non-injectable treatment, or adolescence are excluded. Editorials, notes, letters, case reports, and reviews are excluded from the study. Any study that the full text was unavailable was excluded from the review. Studies that were unavailable in English were excluded. Period of publication was filtered for studies released post the year 2000 for the studies included in the result section.

### Search strategy

A comprehensive search strategy was developed for each database (MEDLINE, Embase, Scopus and web of science) with help from the medical librarian. The search strategy included the use of various keywords using the “And” and “Or” search features. Keywords included: Hip Osteoarthritis, Corticosteroid, Local anaesthetic, saline, Hyaluronic acid, platelet rich plasma (PRP), stem cells, bone marrow aspirate concentrate (BMAC). The studies were then narrowed down using the inclusion and exclusion criteria. Stem cells, PRP and BMAC are not involved in the study due to a lack of papers to fit the inclusion criteria. Searches took place from 08/01/24 – 22/01/24.

MEDLINE search strategy for example used the different keywords mentioned previously—Hip Osteoarthritis, Corticosteroid, Local anaesthetic, saline, Hyaluronic acid, platelet rich plasma (PRP), stem cells, bone marrow aspirate concentrate (BMAC). “And” and “Or” search methods were used with the keywords to maximise the number of papers found. The keyword hip osteoarthritis was set as an “and” the different treatments were set as “Or.” The exclusion criteria was added in to exclude papers that didn’t fit the criteria. The remaining papers found in the search were assessed individually to see if they fit the inclusion criteria. The papers that passed then had the full text reviewed and any that didn’t suit were excluded. Full search string ( TITLE-ABS-KEY ( "hip osteoarthritis") AND TITLE-ABS-KEY ( corticosteroid) OR TITLE-ABS-KEY ( "platelet rich plasma") OR TITLE-ABS-KEY ( "stem cell") OR TITLE-ABS-KEY ( "bone marrow aspirate concentrate") OR TITLE-ABS-KEY ( "local anesthetic") OR TITLE-ABS-KEY ( saline) OR TITLE-ABS-KEY ( "hyaluronic acid") OR TITLE-ABS-KEY ( prp) OR TITLE-ABS-KEY ( bmac)). The articles had to compare either corticosteroids or an alternative treatment to another treatment such as placebo, to provide a measurable outcome that could be compared statistically and within the meta-analysis. Ideally the articles would show clinical improvement and reduced pain or other symptoms.

### Study selection and data extraction

The titles and abstracts of articles obtained were screened with articles not meeting inclusion criteria being removed. The remaining publications were further analysed to ensure quality and for final inclusion in the review. Each included trial required at least one measurement of pain – WOMAC or VAS. Relevant data was extracted on prospective trial methodology – study participants, location of study, interventions, injection dosage, study design; follow up; comparator; blinding; outcome measures and results. 2 reviewers (EK and NH) took part in the selection process, one author (EK) worked independently screening the titles and abstracts of publications, which was then independently reviewed by a second screener, NH. Both authors came together to screen remaining full texts for eligibility and excluded any unsuitable. An unsuccessful attempt was made to contact one of the authors (Qvistgaard et al. [41]) to ask for detailed results as those provided were unsuitable for use within the meta-analysis.

**Fig. 1:**
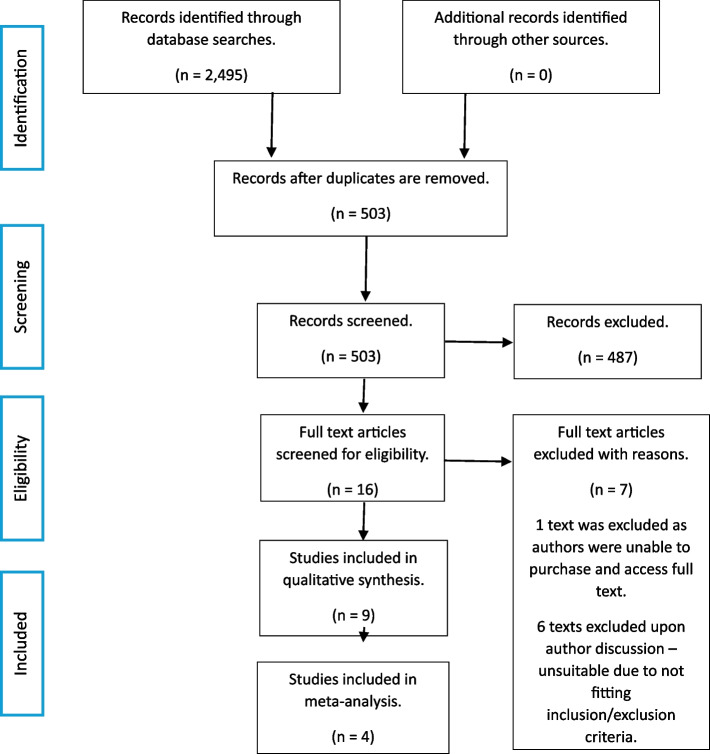
Summary of search results and trial selection

The search yielded 2495 studies, 1992 of which were duplicates and when removed left 503 studies. Screening was completed for the title and abstracts of the remaining 503 studies with 487 being excluded. Full texts were identified for the remaining 16 texts. Out of the 16, 9 trials were included in the final analysis with 4 being used within the meta-analysis, all of which were published between 2004 and 2022. Further details of the literature search are demonstrated in Fig. 1 below.

### Statistical analysis

To create the forest plots for the meta-analysis the software RevMan was used. The data included within the tables in the results section was extracted from the various studies included in the review. The data was then used within RevMan to create the forest plots allowing assessment of statistical significance within the data. The data present in the tables were statistical results produced by the different studies that were assessed within the review, which was extracted, and the various tables were created.

To manage heterogeneity studies were assessed using risk of bias tools to exclude poor quality studies from the results. Studies were separated into groups for the meta-analysis to reduce heterogeneity as much as possible, this included only comparing the same intervention type, pain scale and time frame within each individual forest plot. However, the ¾ plots showed a high heterogeneity, but this is possibly due to the lack of studies to match the criteria of each comparison.

Primary outcome:

- Change in pain scores using the WOMAC or VAS scales at 2-4 months post intervention.

Secondary outcome:

- Outcomes at other time periods such as 1, 6- and 12-months post intervention where available.
- Change in joint mobility and function post intervention.
- Effects of volume

### Quality assessment

Each article was assessed for risk of bias using the Risk of Bias 2 tool [42] with guidance from the Cochrane Handbook of Systematic Reviews of Interventions (Table 1) [43]. The articles were also assessed using the Joanna Briggs Institute (JBI) Checklist for Randomised Controlled Trials and is assessed in Table 2 [44]. For total JBI scores high quality studies were identified based on a score higher than 70%, those between 50 and 70% are considered medium quality and under 50% are considered low quality. Meta-analysis forest plots were created using means, standard deviation and standard error margins provided in the included publications results and created using the software Review Manager.

**Table 1:** Risk of Bias Evaluation Table

**Risk of Bias Checklist**	**Young et al. (2012)** [45]	**Qvistgaard et al. (2006)** [41]	**Paskins et al. (2022)** [46]	**Kullenberg et al. (2004)** [47]	**Lambert et al. (2007)** [48]	**Aksoy et al. (2022)** [49]	**De Rezende et al. (2020)** [50]	**Jurgensmeier et al. (2021)** [51]	**Spitzer et al. (2010)** [52]
Random sequence generation (selection bias)	Low risk – block randomization	High risk	Low risk—parallel group, three arm randomized controlled trial	Low risk	Low risk—Randomized placebo-controlled trial	High risk	Low risk—prospective, randomized clinical trial	Low risk—randomized controlled trial	Low risk – randomized comparative trial
Allocation concealment (selection bias)	Low risk	Unclear	Low risk	Low risk	Low risk—double blind	Low risk	Low risk – double blind	Low risk – double blind	Low risk – double blind
Blinding of participants and researchers (performance bias)	High risk – patients blinded, radiologist injecting treatment wasn’t	Low risk	High risk – researchers unblinded	High risk – researchers unblinded	Low risk	High risk – researchers unblinded	Unclear risk – unclear if researchers are blinded	Low risk	Low risk
Blinding of outcome assessment (detection bias)	Unclear risk	Low risk	Low risk	Low risk	Low risk	High risk	Low risk	Low risk	Low risk
Incomplete outcome data (attrition bias)	High risk – patients lost to follow up without reason	Low risk	Low risk	High risk – patients lost to follow up without reason	High risk – patients lost to follow up without reason	High risk –patients lost to follow up	Low risk	High risk – patients lost to follow up without reason	High risk – patients lost to follow up
Selective reporting (reporting bias)	Low risk	Low risk	Low risk	Low risk	Low risk	Low risk	Low risk	Low risk	Low risk
Other bias	Low risk	Low risk	Low risk	Low risk	Low risk	Low risk	Low risk	Low risk	Low risk
Overall Assessment	High risk	Low risk	Low risk	High risk	Low risk	High risk	Low risk	Low risk	Low risk

**Table 2:** JBI Critical Appraisal Checklist

**JBI Critical Appraisal Checklist**	**Young et al. (2012)** [45]	**Qvistgaard et al. (2006)** [41]	**Paskins et al. (2022)** [46]	**Kullenberg et al. (2004)** [47]	**Lambert et al. (2007)** [48]	**Aksoy et al. (2022)** [49]	**De Rezende et al. (2020)** [50]	**Jurgensmeier et al. (2021)** [51]	**Spitzer et al. (2010)** [52]
Was true randomization used for assignment of participants to treatment groups?	Yes – 1	No – 0	Yes – 1	Yes – 1	Yes – 1	Unclear – 0	Yes – 1	Yes – 1	Yes – 1
Was allocation to treatment groups concealed?	Yes – 1	Unclear – 0	No – 0	Yes – 1	Yes – 1	Yes – 1	Yes – 1	Yes – 1	Yes – 1
Were treatment groups similar at the baseline?	Yes – 1	Yes – 1	Yes—1	Yes – 1	Yes – 1	Yes – 1	Yes – 1	Yes – 1	Yes – 1
Were participants blind to treatment assignment?	Yes – 1	Yes – 1	Yes – 1	Yes – 1	Yes – 1	Yes – 1	Yes – 1	Yes – 1	Yes—1
Were those delivering treatment blind to treatment assignment?	No—0	Yes – 1	No – 0	No – 0	Yes – 1	No – 0	Unclear – 0	Yes – 1	No – 0
Were outcomes assessors blind to treatment assignment?	Unclear – 0	Yes – 1	Yes – 1	Yes – 1	Yes – 1	No – 0	Yes – 1	Yes – 1	Yes – 1
Were treatment groups treated identically other than the intervention of interest?	Yes – 1	Yes – 1	Yes – 1	Yes – 1	Yes – 1	Yes – 1	Yes – 1	Yes – 1	Yes -1
Was follow up complete and if not, were differences between groups in terms of their follow up adequately described and analyzed?	No – 0	Yes – 1	Yes – 1	Unclear – 0	No – 0	No – 0	Yes – 1	No – 0	No – 1
Were participants analyzed in the groups to which they were randomized?	No – 0	Yes – 1	No – 0	Yes – 1	No – 0	No – 0	No – 0	No – 0	Yes – 1
Were outcomes measured in the same way for treatment groups?	Yes – 1	Yes – 1	Yes – 1	Yes – 1	Yes – 1	Yes – 1	Yes – 1	Yes – 1	Yes – 1
Were outcomes measured in a reliable way?	No – 0 WOMAC completed via phone	Yes – 1	No – 0 self-reported pain	Yes – 1	Yes – 1	Yes – 1	Yes – 1	Yes – 1	Yes—1
Was appropriate statistical analysis used?	Yes – 1	Yes – 1	Yes – 1	Yes – 1	Yes – 1	Yes – 1	Yes – 1	Yes – 1	Yes – 1
Was the trial design appropriate, and any deviations from the standard RCT design (individual randomization, parallel groups) accounted for in the conduct and analysis of the trial?	Yes – 1	Yes – 1	Yes – 1	Yes – 1	Yes – 1	No – 0 comparative study, high risk of bias	Yes – 1	Yes – 1	Yes – 1
Total Score	8/13 62%	11/13 85%	9/13 69%	11/13 85%	11/13 85%	7/13 53%	11/13 85%	11/13 85%	11/13 85%
Include / Exclude	Include	Include	Include	Include	Include	Include	Include	Include	Include

## Results

### Search Results

The studies included, were of a good standard of quality overall. 6/9 studies also fit the criteria for having a low risk of bias, showing reliability. However, there was a lack of papers for other injection therapies such as stem cells which has limited the study and meta-analysis. The 6 studies showed a low risk of bias whilst also being determined to be off a high quality under the JBI critical appraisal, with the remaining 3 high risk studies determined to be of medium quality.

### Risk of Bias

Using Cochrane risk of bias evaluation (Table 1) 6 studies achieved a low risk of bias, while 3 studies including Young [45], Kullenberg [47] and Aksoy [49]were shown to be at a high risk of bias. These studies were presumed high risk due to a lack of blinding of researchers and the loss of follow up with patients without implementing intention to treat analysis (ITT). Aksoy [49] is a retrospective study and had no mention of randomisation leading to increased risk of bias. However, all studies faced losing patients to follow up and only Qvistgaard [41], Paskins [46] and De Rezende [50] incorporated ITT, which indicates a risk of bias within outcome measures. Using JBI appraisal scores 6 studies were shown to be high quality and 3 of medium quality (Table 2). None of the papers appeared to be subject to sponsorship bias.

### Study characteristics

The summary of the characteristics of the nine trials are presented in Table 3. The studies included 1083 adult patients, 532 received CS, 163 received placebo and 236 received HA only. Mean patient age was 63 years and mean BMI was 29 across 5 studies [46, 49–52]. Only 4 studies [41, 49, 52, 50] used Kellgren Lawrence grading systems to determine radiology severity of the hip OA patients, with Kullenberg using the Ahlback method. Kullenberg also used the 5 grade Katz and Akpom scale as a measure of function and a goniometer as a measure of joint range of movement [47]. 3 studies compared CS to placebo [46–48], 2 compared CS to HA [49, 52], 1 compared CS to placebo and HA [41], 2 compared injection volume [45, 50] and 1 compared CS to ketorolac [51].

**Table 3:** Summary of characteristics in included papers

Study	Sample	Dropouts	Study Design	Population	Intervention	Follow up period	Outcome Measures
**Young et al. (2012)** [45]	121 referred 110 participated 65% female 55 received triamcinolone + bupivacaine, 55 received sterile water + triamcinolone + bupivaciane	11 excluded 8 withdrawn	Block Randomisation	UK patients Age 20–93	1 injection per group, received once Intra articular fluoroscopically guided 40 mg triamcinolone + 2 mg bupivacaine (3 ml), 40 mg triamcinolone + 2 mg bupivacaine + 6 ml sterile water (9 ml)	3 months	WOMAC and Oxford pain chart
**Qvistgaard et al. (2006)** [41]	185 referred 81 excluded 101 included 64% female 33 received HA 36—Saline 32—CS	81 excluded 6 lost to follow up 7 discontinued 3 withdrew consent	Double blind, randomized controlled trial with three-armed parallel group design	Denmark patients Age 28–88	IA injection, ultrasound guided 1—one injection with 1 mL (40 mg Depo-medrol®) methylprednisolone CS followed by two sham injections 2—three injections of 2 mL HA (Hyalgan®) 3—three intra-articular injections of 2 mL saline water In all cases, including the sham injections, 1 mL of 1% lidocaine was added to the syringe The three intra-articular injections were given at 14 days interval Kellgren Lawrence grade 1–4	3 months	Primary outcome—‘pain on walking’ VAS scale Secondary outcome—‘pain at rest’ VAS, Lequesne score, and WOMAC
**Paskins et al. (2022)** [46]	199 patients 57% female 66—IA CS + LA 66—LA + education 67—Education	16 withdrew (4 no reason given)	Hip Injection Trial (HIT), a pragmatic, three arm, parallel group, single blind, randomised controlled trial	UK patients Age > 40	IA injection, ultrasound guided 67—receive advice and education (best current treatment (BCT)) 66 – BCT + 40 mg/ml triamcinolone and 4 ml 1% lidocaine 66 – BCT + 5 ml 1% Lidocaine	6 months	Main—NRS of hip pain intensity Secondary – pain, stiffness and physical function (WOMAC)
**Kullenberg et al. (2004)** [47]	80 patients 40—IA CS 40—LA	Not stated, all LA group withdrew at 12 weeks due to lack of results	Single Blind, prospective study	Swedish patients Group 1 – mean age = 67.3 ± 7.7 years Group 2—72.7 ± 6.4 years	IA injection, fluoroscopically guided 2 Groups 1 – N = 40 – CS (80 mg triamcinolone acetonide) 2 – N = 40 – LA (1% mepivacaine) Ahlback grade 2 or worse Function – 5 grade Katz and Akpom scale Movement—Goniometer	6 months	Pain measured by VAS before and after injection with reference to pain at rest and on bearing weight
**Lambert et al. (2007)** [48]	211 referred 52 participated 59% female 21 – placebo 31—CS	159 excluded 33 withdrew (8 lost to follow up)	Randomized double blind placebo-controlled trial	Canadian patients Age > 40 Placebo – age—56.9 ± 11 CS – age—65.6 ± 11	Fluoroscopically guided IA injection 1—placebo (10 mg bipuvicaine, 2 ml saline) (n = 21) 2—corticosteroid treatment (10 mg bipuvicaine, 40 mg triamcinolone hexacetonide) (n = 31)	6 months	Primary outcome—pain improvement response using WOMAC Secondary outcomes – WOMAC scores on stiffness and function, and VAS scores SF-36 compartment scores
**Aksoy et al. (2022)** [49]	137 referred 95 participants 37% females 48 – CS 47—HA	29 excluded 13 lost to follow up	Retrospective comparative study, single blind	Turkish patients CS – age—64.54 ± 9.70, HA – age—62.53 ± 13.43,	Radiologically guided vs blinded IA injections Group 1 – CS triamcinolone Group 2 – Sodium hyaluronate 88 mg/4 ml Stage 2–4 Kellgren-Lawrence (KL) grade	12 months	Primary—WOMAC pain scores, Secondary – KL grade
**De Rezende et al. (2020)** [50]	536 assessed 82 included 80% female 19—Group 0 – lavage + triamcinolone + lidocaine 19—Group 1 – lavage, triamcinolone + hylan G-F20 + lidocaine 22—Group 2 – lavage + triamcinolone + hylan G-F20 + lidocaine 22—Group 3 – lavage + triamcinolone + hylan G-F20 + lidocaine	454 excluded 2 withdrew	Double blind, prospective, randomized clinical trial	Brazilian patients Mean age—62	Intra articular injection Group 0 – lavage and triamcinolone (1 ml) and 2 ml of lidocaine Group 1 – lavage, triamcinolone and 2 ml hylan G-F20 and 2 ml of lidocaine Group 2 – lavage, triamcinolone, and 4 ml of hylan G-F20 and 2 ml of lidocaine Group 3 – lavage, triamcinolone and 6 ml of hylan G-F20 and 2 ml of lidocaine KL Grade 2 + 3 Hip OA	12 months	VAS, range of motion, WOMAC and Lequesne
**Jurgensmeier et al. (2021)** [51]	120 patients (52 hips) 64% of total were female 30—CS 28—Ketorolac	Of 52 hips 6 patients lost to follow up	Double blind, randomized controlled trial	USA patients	Ultrasound guided IA injection Group 1 – 5 ml of 0.5% ropivacaine with 80 mg of triamcinolone Group 2 – 5 ml of 0.5% ropivacaine with 30 mg of Ketorolac Stage 2 KL grade or higher	3 months	Primary – VAS Secondary—HOOS
**Spitzer et al. (2010)** [52]	312 patients 51% Female 156 CS 156 HA	109 discontinued 4 lost to follow up 20 – adverse event 65—Wish to withdraw 4—Non-compliant 16—Other	Double blind, parallel randomised competitive trial	USA patients	Fluoroscopically guided IA injection 2 IA 2 ml injections of hylan G-F 20 Or 1 IA injection of 2 ml methylprednisolone + a sham injection	6 months	Primary – WOMAC pain, function, stiffness and overall Secondary – Kellgren Lawrence, VAS

WOMAC and VAS were the most frequently used outcome score used by 6/9 studies for WOMAC and 5/9 studies for VAS, Lequesne scores were used by 2 studies. Young used the Oxford Pain Chart as a secondary measure [45]. Paskins was the only study to use numerical rating scale as the primary outcome measure [46]. Lambert used the SF-36 method as a secondary method and Jurgensmeier utilised HOOS scores [48, 51]. The nine studies reported outcomes at 1 week (1), 2 weeks (2), 3 weeks (1), 1 month (5), 2 months (2), 3 months (7), 4 months (1), 6 months (5), 12 months (2).

No study included patients with hip IA injections in the previous 3 months, hip co morbidities such as necrosis of the hip, systemic diseases or those who had previously undergone hip surgery. 4 of the studies were in Europe, 3 in North America, 1 in Asia and 1 in South America.

All the studies used similar dosages and preparations of CS, saline and HA, Spitzer and Qvistgaard were the only studies to use more than one injection as seen in Table 4 [41, 52].

**Table 4:** Injection Characteristics

Study	Injection	Date
**Young et al. ** [45]	1 – 40 mg triamcinolone + 2 mg bupivacaine (3 ml), 2 – 40 mg triamcinolone + 2 mg bupivacaine + 6 ml sterile water (9 ml)	2012 – UK
**Qvistgaard et al. ** [41]	1 – 1 × 1 mL (40 mg Depo-medrol®) methylprednisolone CS followed by 2 × sham injections 2 – 3 × 2 mL HA (Hyalgan®) 3 – 3 × 2 mL saline water In all cases, including the sham injections, 1 mL of 1% lidocaine was added to the syringe	2006 – Denmark The three intra-articular injections were given at 14 days interval
**Paskins et al. ** [46]	1 – receive advice and education (best current treatment (BCT)) 2 – BCT + 40 mg/ml triamcinolone and 4 ml 1% lidocaine 3 – BCT + 5 ml 1% Lidocaine	2022 – UK
**Kullenberg et al. ** [47]	1 – CS (80 mg triamcinolone acetonide) 2 – LA (1% mepivacaine)	2004 – Sweden
**Lambert et al. ** [48]	1—placebo (10 mg bupivacaine, 2 ml saline) 2—corticosteroid treatment (10 mg bupivacaine, 40 mg triamcinolone hexacetonide)	2007 – Canada
**Aksoy et al. ** [49]	1 – CS triamcinolone 2 – Sodium hyaluronate 88 mg/4 ml	2022 – Turkey
**De Rezende et al. ** [50]	0 – lavage and triamcinolone (20 mg)(1 ml) and 2 ml of lidocaine 1 – lavage, triamcinolone and 2 ml hylan G-F20 and 2 ml of lidocaine 2 – lavage, triamcinolone, and 4 ml of hylan G-F20 and 2 ml of lidocaine 3 – lavage, triamcinolone and 6 ml of hylan G-F20 and 2 ml of lidocaine	2020 – Brazil
**Jurgensmeier et al. ** [51]	1 – 2 × IA 2 ml injections of hylan G-F 20 2 – 1 × IA injection of 2 ml (40 mg) methylprednisolone + 1 × sham injection	2021 – USA
**Spitzer et al. ** [52]	2 × IA 2 ml injections of hylan G-F 20 Or 1 × IA injection of 2 ml methylprednisolone + 1 × sham injection	2010 – USA Injections 2 weeks apart

### Primary outcomes

Change in WOMAC pain score is presented in Table 5. From the results gathered from the nine studies, five used WOMAC pain scores as an individual measurement. Most of the studies found CS to be superior to placebo and that CS provides significant decrease in pain scores up to one month. One study showed CS to lower pain scores more than HA showing it to be more effective. However, one study, Young shows no decrease in pain scores between comparators but, this is due to the study focusing on hip injection volume rather than comparing injection therapy [45].

**Table 5:** WOMAC pain scores

	**WOMAC Pain Scores**				
	**Time scale**	**Treatments**			
**Study**		**Corticosteroid (CS) 3 ml**		**CS (3 ml) + (6 ml) saline**	
**Young et al** (110 patients) [45]	**Pain (Baseline)**	12.2		12.3	
	**Pain (3 months)**	8.8		8.9	
		**CS**		**Placebo**	
		**Mean, (SD), N**		**Mean, (SD), N**	
**Paskins et al. **(199 patients) [46]	**Pain (Baseline)**	10.7 ± 4.0, 66		10.7 ± 2.8, 66	
	**Pain (2 months)**	7.0 (4.3), 61		8.7 (4.1), 62	
	**Pain (4 months)**	7.9 (4.3), 59		9.1 (4.1), 59	
	**Pain (6 months)**	8.8 (4.3), 56		9.1 (4.1), 60	
		**CS**		**Placebo**	
**Lambert et al. **(52 patients) [48]	**Pain (Baseline)**	310.1 ± 54.6 mm		314.3 ± 76.2	
	**Pain (1 month)**	149.6 ± 113 mm		276.4 ± 129.0	
	**Pain (2 months)**	157.4 ± 127.2 mm		306.5 ± 121.2	
		**CS**		**Hyaluronic Acid**	
**Spitzer et al. **(312 patients) [52]	**Pain (Baseline)**	64.53 ± 0.98		63.40 ± 1.00	
	**Pain (1 month)**	35.04 ± 1.91		45.5 ± 1.97	
	**Pain (6 months)**	48.47 ± 2.47		44.03 ± 2.48	
		**Group 0—CS**	**Group 1—CS + HA**	**Group 2—CS + HA**	**Group 3—CS + HA**
**De Rezende et al. **(82 patients) [50]	**Pain (Baseline)**	11.1 (3.3)	10.4 (3.3)	10.5 (4.9)	10.5 (4.4)
	**Pain (1 months)**	6.6 (5.4)	6.0 (3.9)	6.9 (5.2)	6.8 (4.4)
	**Pain (3 months)**	7.1(4.2)	7.9 (3.3)	7.4 (4.4)	7.0 (5.0)
	**Pain (6 months)**	6.9 (4.6)	8.0 (4.7)	6.4 (4.9)	7.9 (4.7)
	**Pain (12 months)**	6.4 (4.1)	7.7 (4.8)	8.0 (5.2)	7.6 (4.7)

The analysis of VAS score results is shown below in Table 6, with 5/9 studies evaluated using VAS. Again, all the studies showed CS to be superior to placebo. Qvistgaard even showed CS to be superior to HA and placebo, they also showed HA to lower pain scores further than placebo [41]. De Rezende shows CS + 4 ml of HA to be the most effective option at reducing pain scores when compared to CS on its own or conjugated with 2/6 ml of HA [50]. CS presented no improvement on pain scores when compared to ketorolac in one study.

**Table 6:** VAS pain scores

	**VAS Pain Scores**				
	**Time scale**	**Treatments**			
**Study**		**CS**	**HA**	**Placebo**	
	**Pain on walking 0-100 mm**				
**Qvistgaard et al** (101 patients) [41]	**Baseline**	45	45	45	
	**Pain (14 days)**	33	37	46	
	**Pain (1 month)**	30	36	40	
	**Pain (3 months)**	36	36	39	
	**Pain at rest 0-100 mm**				
	**Baseline**	25	25	25	
	**Pain (14 days)**	22	23	29	
	**Pain (1 month)**	22	26	25	
	**Pain (3 months)**	23	27	28	
	**0–20 Pain scale**	**CS**		**Placebo**	
**Kullenberg et al. **(80 patients) [47]	**Pain (Baseline)**	12.2 ± 2.2		12.0 ± 1.0	
	**Pain (3 weeks)**	3.8 ± 2.6		12.4 ± 1.8	
	**Pain (3 months)**	7.9 ± 3.9		12.4 ± 1.8	
		**Group 0—CS**	**Group 1—CS + HA**	**Group 2—CS + HA**	**Group 3—CS + HA**
**De Rezende et al. **(82 patients) [50]	**Pain (Baseline)**	63.8 (21.5)	68.2 (21.8)	55.8 (31.4)	69.2 (20.9)
	**Pain (1 month)**	35.5 (33.4)	31.9 (22.0)	28.4 (27.2)	44.0 (34.3)
	**Pain (3 months)**	44.3 (31.7)	43.3 (21.9)	40.1 (29.3)	43.0 (29.7)
	**Pain (6 months)**	46.2 (28.0)	49.9 (30.0)	37.6 (28.9)	43.0 (31.4)
	**Pain (12 months)**	40.3 (34.4)	48.4 (27.0)	40.8 (27.8)	46.6 (27.7)
		**CS**		**Ketorolac**	
**Jurgensmeier et al. **(120 patients) [51]	**Pain (Baseline)**	5.42		5.27	
	**Pain (3 months)**	4.31		4.19	

### Secondary outcomes

Results of overall, stiffness and function WOMAC scores are presented in Table 7. All studies within the systematic review showed evidence of CS providing improvements in stiffness, function and overall WOMAC when compared to placebo until 2 months. After 2 months there was not a significant difference in scores. Studies analysing CS vs HA found dissimilar in scores up to one month. After one-month results showed no significant difference except Qvistgaard who showed no significant difference at any time [41]. De Rezende showed significant improvements in scores all around, however there was no significant differences between groups and injection volume [50]. Young also showed no difference in improvements between injection volumes [45].

**Table 7:** Further WOMAC scores—Overall, stiffness and function

	**Secondary WOMAC Scores**				
	**Time scale**	**Treatment**			
**Study**		**Corticosteroid (CS) 3 ml** **40 mg triamcinolone + 2 mg bupivacaine (3 ml)**		**CS (3 ml) + (6 ml) saline**	
**Young et al. **(110 patients) [45]	**Stiffness – Baseline**	5		5.2	
	**3 months**	3.9		3.7	
	**Function – Baseline**	42.1		44.3	
	**3 months**	33.8		34	
		**CS** **40 mg methylprednisolone**	**Hyaluronic Acid** **2 mL HA (Hyalgan®)**	**Placebo** **2 mL saline**	
**Qvistqaard et al** (101 patients) [41]	**Overall Baseline**	39	39	39	
	**14 days**	33	34	38	
	**1 month**	32	33	36	
	**3 months**	33	35	34	
		**CS** **Single dose of triamcinolone**		**HA** **Sodium hyaluronate 88 mg/4 ml**	
**Aksoy et al. **(95 patients) [49]	**Baseline Overall**	67.94 ± 9.01		71.64 ± 9.05	
	**3rd-month**	58.73 ± 7.95		61.32 ± 5.75	
	**6th-month**	60.92 ± 6.68		63.55 ± 8.04	
	**12th-month**	67.75 ± 8.96		70.53 ± 7.28	
		**CS** **40 mg/ml triamcinolone and 4 ml 1% lidocaine**		**Placebo** **5 ml 1% Lidocaine**	
**Paskins et al. **(199 patients) [46]	**Overall Baseline**	50.2 (14.8) 65		50.7 (13.0) 65	
	**2 months**	34.2 (20.3) 61		41.4 (19.2) 62	
	**4 months**	38.3 (20.7) 59		43.9 (18.5) 56	
	**6 months**	41.8 (20.8) 55		44.0 (19.4) 59	
	**Stiffness (Baseline)**	4.6 (1.4) 65		4.6 (1.5) 65	
	**2 months**	3.2 (1.9), 63		3.7 (1.7), 63	
	**4 months**	3.7 (1.9), 60		3.8 (1.8), 60	
	**6 months**	3.7 (1.7), 56		3.8 (1.8), 59	
	**Function (Baseline)**	35.0 (11.6) 65		35.4 (10.9) 65	
	**2 months**	23.8 (15.0), 62		29.1 (14.3), 63	
	**4 months**	26.7 (15.1), 60		31.3 (13.5), 59	
	**6 months**	28.8 (15.2), 57		31.0 (14.3), 59	
		**CS** **10 mg bipuvicaine, 40 mg triamcinolone hexacetonide**		**Placebo** **10 mg bipuvicaine, 2 ml saline**	
**Lambert et al. **(52 patients) [48]	**Stiffness (Baseline)**	137.4 ± 33.0		124.5 ± 37.7	
	**Stiffness (1 month)**	79.6 ± 57.3		119.8 ± 43.8	
	**Stiffness (2 months)**	75.6 ± 58.1		126.8 ± 48.4	
	**Function (Baseline)**	969.3 ± 167.8		970.9 ± 254.5	
	**Function (1 month)**	516.0 ± 388.1		897.4 ± 369.3	
	**Function (2 months)**	538.5 ± 402.0		949.1 ± 350.4	
		**CS** **2 ml methylprednisolone + a sham injection**		**HA** **2 ml hylan G-F 20 × 2**	
**Spitzer et al. **(312 patients) [52]	**Stiffness (Baseline)**	65.06 ± 1.48		66.12 ± 1.52	
	**Stiffness (1 month)**	38.73 ± 1.83		50.22 ± 1.89	
	**Stiffness (6 months)**	51.36 ± 2.45		50.6 ± 2.47	
	**Function (Baseline)**	63.03 ± 1.37		64.42 ± 1.40	
	**Function (1 month)**	36.46 ± 1.73		46.23 ± 1.79	
	**Function (6 months)**	51.5 ± 2.3		50.62 ± 2.32	
	**Overall (Baseline)**	63.26 ± 1.24		64.27 ± 1.27	
	**Overall (1 month)**	36.28 ± 1.69		46.09 ± 1.75	
	**Overall (6 month)**	51.26 ± 2.29		49.57 ± 2.32	
		**Group 0** **lavage and triamcinolone (1 ml) and 2 ml of lidocaine**	**Group 1** **lavage, triamcinolone and 2 ml hylan G-F20 and 2 ml of lidocaine**	**Group 2** **lavage, triamcinolone, and 4 ml of hylan G-F20 and 2 ml of lidocaine**	**Group 3** **lavage, triamcinolone and 6 ml of hylan G-F20 and 2 ml of lidocaine**
**De Rezende et al. **(82 patients) [50]	**Stiffness—Baseline**	4.4 (1.8)	4.1 (1.8)	4.0 (2.3)	4.9 (2.0)
	**One Month**	2.7 (2.2)	2.4 (1.9)	2.8 (2.3)	3.2 (2.4)
	**Three Months**	3.0 (2.1)	2.7 (1.4)	2.5 (2.2)	3.4 (2.4)
	**Six Months**	2.8 (2.2)	3.2 (2.2)	2.1 (2.2)	3.6 (2.3)
	**Twelve Months**	3.4 (2.0)	2.8 (2.1)	2.8 (2.0)	3.6 (2.3)
	**Function—Baseline**	42.1 (8.5)	36.2 (2.7)	35.2 (13.6)	40.6 (9.8)
	**One Month**	22.6 (17.9)	24.7 (14.3)	24.5 (14.8)	27.6 (15.4)
	**Three Months**	24.6 (12.7)	25.7 (13.9)	26.9 (13.9)	27.6 (16.5)
	**Six Months**	28.4 (13.2)	29.4 (16.0)	24.9 (17.7)	29.6 (13.9)
	**Twelve Months**	26.6 (13.2)	28.2 (15.7)	27.4 (16.7)	31.3 (16.3)
	**Total—Baseline**	56.8 (12.2)	50.3 (16.2)	49.0 (19.5)	56.2 (16.6)
	**One Month**	31.8 (24.9)	33.1 (19.1)	33.0 (22.7)	37.6 (21.1)
	**Three Months**	34.6 (17.4)	36.3 (17.5)	36.8 (19.7)	38.4 (23.2)
	**Six Months**	38.1 (18.6)	40.6 (22.3)	33.3 (24.2)	41.0 (19.7)
	**Twelve Months**	36.4 (18.0)	38.7 (21.5)	38.3 (23.1)	42.5 (22.5)

### Meta-analysis

**Fig. 2:**
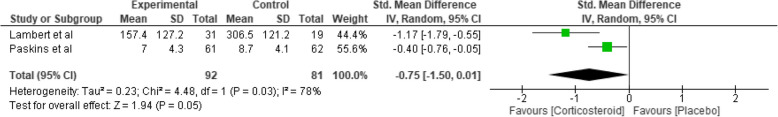
WOMAC pain score forest plot at 2 months post injection [46, 48]

**Fig. 3:**
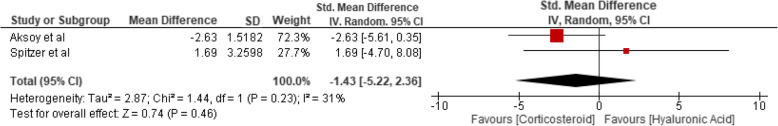
Overall WOMAC score forest plot at 6 months post injection [49, 52]

**Fig. 4:**
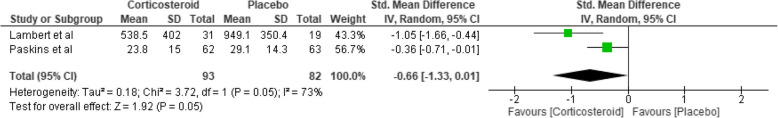
WOMAC function score forest plot at 2 months post injection [46, 48]

**Fig. 5:**
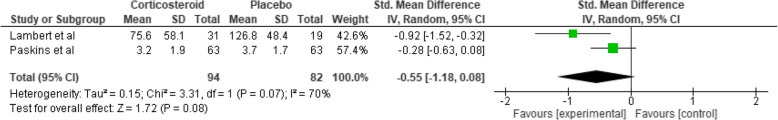
WOMAC stiffness score forest plot at 2 months post injection [46, 48]

The meta-analysis is shown in Figs. 2, 3, 4, and 5 shown below. Figure 2 presents WOMAC pain scores at 2 months and does not show a statistical significance pointing towards CS being favoured over placebo injections. Figure 3 presents overall WOMAC at 6 months presenting statistically insignificant results, slightly on the side of favouring corticosteroids. Figure 4 compares WOMAC function at 2 months post injection and again doesn’t show statistical significance. Figure 5 compares WOMAC function and again shows no statistical significance for CS. Figure 2 and 4 present a p value of 0.05 which is just outside of statistical significancy, more trials being included within the analysis could lead to more accurate and potent statistical significance.

## Discussion

### Summary of findings

The changes in pain score for WOMAC is presented in Table 5. 2 studies [46, 48] show significant decreases in pain scores for corticosteroids when compared to placebo. At 2 months post injection Lambert showed a massive decrease on pain with a 49.2% decrease while placebo only showed a 2.5% decrease [48]. Paskins also presented a decrease in pain score, showing improvement with the use of corticosteroids compared with the use of placebo [46]. However, 1 study [45] presented no improvement between comparators but this is due to the study focusing on hip injection volume. Spitzer compared CS to HA, while both injections showed significant improvement on pain, CS provided lower pain scores than HA at one month [52]. Although, both injections presented similar results at 6 months [52], showing CS short length of action while HA shows more consistency. De Rezende presented great improvement on pain scores, however, the analysis focused on HA volume and improvement with CS conjugation therefore the data was unable to be used within the meta-analysis and unable to be determined if it was statistically significant [50]. Overall, most of the studies that used the WOMAC pain scale found that CS and HA provided improved pain relief in the short term. No clear outlier was found in the results, only that in the very short term (1 month) CS provided better outcomes. The results also found CS to show improvements in pain compared to placebo.

The analysis of VAS score results are shown in Table 6. Qvistgaard compared CS to HA and placebo with pain on walking and at rest [41]. The results showed again that CS is superior showing improved pain scores on HA and placebo especially at early intervals such as 14 days and one month with pain on walking. HA scores were better than placebo but only matched CS at the 3-month mark (Pain—walking), however according to some authors CS effect only last 6 weeks [14]. However, with pain at rest both CS and HA presented little improvement on pain scores. Placebo also presented higher pain scores at 14 days and at 3 months for pain at rest. Kullenberg presented significant improvements on pain scores at 3 weeks with CS compared to placebo however, by 3 months pain scores rose showing the lessening of CS effect with time [47]. De Rezende interestingly showed that CS + 4 ml dose of HA presented the best impact compared to the other groups, lowering pain scores significantly from baseline at 1 month [50]. All groups showed an effect at 12 months. Jurgensmeier showed no significant difference between CS and ketorolac [51]. The studies using VAS scale also found improvements in the use of CS and HA compared to placebo, however, the results were inconclusive as some studies showed placebo pain scores to be similar to the treatment, while another study didn’t use a placebo comparator.

Overall WOMAC, function and stiffness scores are presented in Table 7. 2/3 studies comparing overall WOMAC scores of CS to HA found no significant difference in scores [41, 49]. Qvistgaard et al. [41] compared CS to HA and placebo and presented little decrease compared to placebo. Spitzer presented CS and HA as significantly decreasing overall scores at 1 month compared to baseline, although CS was significantly lower than HA [52]. Paskins compared CS to placebo presenting significant improvement for CS at baseline compared at 2 and 4 months [46]. Lambert compared CS to placebo with significant improvements in both stiffness and function scores vs placebo and baseline [48]. De Rezende showed improvements in all groups from baseline to 12 months post injection, with no groups results being significantly better than another [50]. Again, inconclusive results were found with some studies showing similar results between therapies and placebo while others found the injections (CS and HA) to show improvements.

### Meta-Analysis review

Previous literature appears different to the meta-analysis results showing CS to be statistically favourable over placebo at early and later time periods regarding all WOMAC scores [14] as the meta-analysis showed statistically insignificant results between the 2 injections (*p* values > 0.05). Data regarding CS vs HA is inconsistent, some papers show no difference between the two injections [53], some show CS to be superior [54, 55], while others show HA to be superior particularly over a longer period [54–56]. Figure 3 provides statistically insignificant results, showing no favour for either CS or HA at 6 months in overall WOMAC scores. This result agrees with previous literature, showing the declining efficacy of CS with time as at 1 month it would be superior to HA [53]. Figures 2 and 4 verge on statistical significance with a p value of 0.05 each, the inclusion of more trials when further trials are published may push the results towards significance. This would then follow previous literature seen in knee OA where CS are supported as the more effective treatment [57]. The forest plots also compare therapies at 2 and 6 months which is past the effective timepoints for CS treatment therefore having a possible negative effect on p values and significance.

### Comparison with previous literature

Many of the results agree with previous literature, for example IA CS are effective for short term pain relief and the most effective time frame appeared to stay around the 1–2-month mark in the studies that analysed that time frame [41, 46–48, 50, 52]. The time frame of 1–2 months being the effective range of CS injections is backed up by various authors [14, 15]. The included studies also showed that CS injections provided some pain relief at periods past 2 months, to a lesser extent and is backed up by further studies [57, 58].

The results agree with previous literature regarding CS vs HA, with little differences being seen at the 6-month mark in any of the studies and in previous studies [53]. However, many articles conclude that over a longer period (> 2 months), HA is superior to CS [54–56]. Although, this discrepancy could be down to the different type of joints as the studies showing HA to be superior long term are studies on the knee. Results gathered from the studies show that CS was more effective in all methods of scoring compared to HA in short term analysis (1 month), agreeing with previous studies [54, 55].

The studies had no withdrawals due to major/unexpected effects, most of the studies experienced minor side effects such as allergic reaction, damage to cartilage, pain, and hot flushes etc. This agrees with literature as CS are known to cause a low incidence of adverse effects. However, if used improperly with high doses and over long durations results can be bad causing long term damage and deterioration of the joints condition [17, 59].

Considering the results, alternatives for CS need to be assessed due to their ineffectiveness and a more effective treatments need to be considered. There are multiple options that could provide improved results on CS such as stem cells, platelet rich plasma and bone marrow aspirate concentrate. These therapies have shown promise in providing improved outcomes in OA treatment and longer periods of effect than CS but further research and comparison to CS is needed [60–65].

### Strengths and Limitations

The study contains several strengths. For the papers included, the majority have been identified as having low risk of bias, showing neutral bias while using effective and blinded techniques for the trials. The paper takes a systematic approach for study analysis using quality assessment tools to validate results and studies included. The use of standard outcome measurements such as WOMAC and VAS between studies was ideal for comparisons. The results show a strong correlation for CS being a superior treatment than placebo/saline injections. Under the JBI critical appraisal checklist none of the studies included were determined to have been of bad quality, the majority (6 papers) were considered high quality and the rest (3 papers) of medium quality.

The study contains minor limitations, primarily stemming from a lack of controlled trials comparing CS to other therapies in hip osteoarthritis. The paper originally was to include other IA therapies such stem cells and platelet rich plasma, but searches found no papers comparing said injections to IA CS. The lack of comparable papers restricted the paper to mostly comparing CS to placebo rather than possible alternative injection therapies as first intended. The meta-analysis is also considerably limited due to the included papers not lining up with follow up times, the use of different scoring methods. At most 2 papers could be used within the forest plots for analysis and even then, the papers didn’t provide enough for VAS score comparison, which is a part of the primary outcome. 2 of the studies did not use a saline or placebo comparator but focused on injection volume, thus limiting their analysis to secondary outcomes [45, 50]. Due to the lack of comparison poor, insignificant results were obtained as a much larger analysis is needed to properly quantify the most effective therapy. The factors limiting the meta-analysis to a small sample base may also have contributed to the analysis disagreeing with current literature showing CS to be superior to placebo, however the meta-analysis was unable to compare outcome measures < 2 months. Therefore, its unknown if CS produce statistically significant improvements at < 2 months post injection. Current research is not at a sufficient point to complete a significant analysis especially to fit criteria that provides a strong, reliable, and unbiased review. A few of the papers were deemed at a high risk of bias, therefore making it difficult to trust the outcomes of their trials. For example, Aksoy [49] is used within Fig. 3 and presented CS as more favourable than HA at 6 months, disagreeing with previous literature making it difficult to trust the results. Furthermore, De Rezende and Young et al., compare injection volume, with both lacking a control, further limiting its use case and the reliability of the results [50, 45]. Studies varied in the age of patients included and some contained considerable differences in sex distribution with the majority containing 60% or more females. Another limitation is possible publication bias with few studies using intention to treat analysis and having patients lost to follow up could’ve led to biased results or a lack of transparency. The limited number of studies and lack of correlation with follow ups and outcome measurement methods was the major limiter for the study. The meta-analysis comparison at 2 and 6 months is past the effective timepoints for CS treatment potentially having negative effects on results as it isn’t analysing CS within its effective range.

### Clinical Implications

Results from the systematic review suggest IA CS as the standout injection therapy similar to previous literature. However, the meta-analysis showed statistically insignificant improvement in the use of CS vs placebo from 2 months onwards post-injection, disagreeing with previous literature in this area [14, 15, 57, 58]. This indicates the urgent need for developing and evaluating new injection therapies to help improve the symptomatic treatment of OA and thus reduce the morbidity associated with this condition. The lack of trials containing newer therapies such as stem cells within hip OA limited possible findings.

## Conclusion

The meta-analysis indicates that CS injections don’t provide a statistically significant improvement when compared to placebo and HA even at 2 months. Therefore, the need to develop other injection therapies to help improve the management and lives of people living with OA is urgent. Further trials are needed to assess other options to CS, especially due to their apparent short period of efficacy and reported associated side effects.

## Data Availability

All data is freely available within the manuscript.

## Appendix 1

### PRISMA Systematic Review Guidelines

**Table:**

Section and Topic	Item #	Checklist item	Reported (Yes/No)	Location where item is reported
**TITLE**			Pg—1	
Title	1	Identify the report as a systematic review	Yes	Pg 1
**ABSTRACT**			Pg – 3	
Abstract	2	See Table 2 for abstract guidelines		Pg 3
**INTRODUCTION**			Pg – 4–6	
Rationale	3	Describe the rationale for the review in the context of existing knowledge	Yes	Pg 4–6
Objectives	4	Provide an explicit statement of the objective(s) or question(s) the review addresses	Yes	Pg 6
**METHODS**			Pg – 6–15	
Eligibility criteria	5	Specify the inclusion and exclusion criteria for the review and how studies were grouped for the syntheses	Yes	Pg 7
Information sources	6	Specify all databases, registers, websites, organisations, reference lists and other sources searched or consulted to identify studies. Specify the date when each source was last searched or consulted	Yes	Pg 7
Search strategy	7	Present the full search strategies for all databases, registers, and websites, including any filters and limits used	Yes	Pg 7–8
Selection process	8	Specify the methods used to decide whether a study met the inclusion criteria of the review, including how many reviewers screened each record and each report retrieved, whether they worked independently, and if applicable, details of automation tools used in the process	Yes	Pg 8
Data collection process	9	Specify the methods used to collect data from reports, including how many reviewers collected data from each report, whether they worked independently, any processes for obtaining or confirming data from study investigators, and if applicable, details of automation tools used in the process	Yes	Pg 8
Data items	10a	List and define all outcomes for which data were sought. Specify whether all results that were compatible with each outcome domain in each study were sought (e.g. for all measures, time points, analyses), and if not, the methods used to decide which results to collect	Yes	Pg 7
	10b	List and define all other variables for which data were sought (e.g. participant and intervention characteristics, funding sources). Describe any assumptions made about any missing or unclear information	Yes	Pg 7–8
Study risk of bias assessment	11	Specify the methods used to assess risk of bias in the included studies, including details of the tool(s) used, how many reviewers assessed each study and whether they worked independently, and if applicable, details of automation tools used in the process	Yes	Pg 10–14
Effect measures	12	Specify for each outcome the effect measure(s) (e.g. risk ratio, mean difference) used in the synthesis or presentation of results	Yes	Pg 10
Synthesis methods	13a	Describe the processes used to decide which studies were eligible for each synthesis (e.g. tabulating the study intervention characteristics and comparing against the planned groups for each synthesis (item #5))	Yes	Pg 10
	13b	Describe any methods required to prepare the data for presentation or synthesis, such as handling of missing summary statistics, or data conversions	Yes	Pg 8
	13c	Describe any methods used to tabulate or visually display results of individual studies and syntheses	Yes	Pg 9
	13d	Describe any methods used to synthesize results and provide a rationale for the choice(s). If meta-analysis was performed, describe the model(s), method(s) to identify the presence and extent of statistical heterogeneity, and software package(s) used	Yes	Pg 8–9
	13e	Describe any methods used to explore possible causes of heterogeneity among study results (e.g. subgroup analysis, meta-regression)	Yes	Pg 8–9
	13f	Describe any sensitivity analyses conducted to assess robustness of the synthesized results	Yes	Pg 8
Reporting bias assessment	14	Describe any methods used to assess risk of bias due to missing results in a synthesis (arising from reporting biases)	Yes	Pg 11–14
Certainty assessment	15	Describe any methods used to assess certainty (or confidence) in the body of evidence for an outcome	Yes	Pg 10–14

**Table:**

Section and Topic	Item #	Checklist item	Reported (Yes/No)	Location where item is reported
**RESULTS**				Pg – 15–31
Study selection	16a	Describe the results of the search and selection process, from the number of records identified in the search to the number of studies included in the review, ideally using a flow diagram	Yes	Pg 9, 15
	16b	Cite studies that might appear to meet the inclusion criteria, but which were excluded, and explain why they were excluded	No	
Study characteristics	17	Cite each included study and present its characteristics	Yes	Pg 15–21
Risk of bias in studies	18	Present assessments of risk of bias for each included study	Yes	Pg 15
Results of individual studies	19	For all outcomes, present, for each study: (a) summary statistics for each group (where appropriate) and (b) an effect estimate and its precision (e.g. confidence/credible interval), ideally using structured tables or plots	Yes	Pg 22–28
Results of synthesis	20a	For each synthesis, briefly summarise the characteristics and risk of bias among contributing studies	Yes	Pg 15–16
	20b	Present results of all statistical syntheses conducted. If meta-analysis was done, present for each the summary estimate and its precision (e.g. confidence/credible interval) and measures of statistical heterogeneity. If comparing groups, describe the direction of the effect	Yes	Pg 28–29
	20c	Present results of all investigations of possible causes of heterogeneity among study results	No	
	20d	Present results of all sensitivity analyses conducted to assess the robustness of the synthesized results	No	
Reporting biases	21	Present assessments of risk of bias due to missing results (arising from reporting biases) for each synthesis assessed	Yes	Pg 15
Certainty of evidence	22	Present assessments of certainty (or confidence) in the body of evidence for each outcome assessed	Yes	22–28
**DISCUSSION**			Pg – 31–35	
Discussion	23a	Provide a general interpretation of the results in the context of other evidence	Yes	Pg 31–33
	23b	Discuss any limitations of the evidence included in the review	Yes	Pg 34–35
	23c	Discuss any limitations of the review processes used	Yes	Pg 34–35
	23d	Discuss implications of the results for practice, policy, and future research	Yes	Pg 34
**OTHER INFORMATION**			Pg – 7/36	
Registration and protocol	24a	Provide registration information for the review, including register name and registration number, or state that the review was not registered	Yes	Pg 7
	24b	Indicate where the review protocol can be accessed, or state that a protocol was not prepared	Yes	Pg 7
	24c	Describe and explain any amendments to information provided at registration or in the protocol	Yes	Pg 7
Support	25	Describe sources of financial or non-financial support for the review, and the role of the funders or sponsors in the review	Yes	Pg 36
Competing interests	26	Declare any competing interests of review authors	N/A	Pg 36
Availability of data, code and other materials	27	Report which of the following are publicly available and where they can be found: template data collection forms; data extracted from included studies; data used for all analyses; analytic code; any other materials used in the review	N/A	Pg 37–41

## Appendix 2

### PRISMA Abstract Guidelines

**Table:**

Section and Topic	Item #	Checklist item	Reported (Yes/No)	Location where item is reported
**TITLE**			Pg – 3	
Title	1	Identify the report as a systematic review	Yes	Pg 3
**BACKGROUND**				
Objectives	2	Provide an explicit statement of the main objective(s) or question(s) the review addresses	Yes	Pg 3
**METHODS**				
Eligibility criteria	3	Specify the inclusion and exclusion criteria for the review	Yes	Pg 3
Information sources	4	Specify the information sources (e.g. databases, registers) used to identify studies and the date when each was last searched	Yes	Pg 3
Risk of bias	5	Specify the methods used to assess risk of bias in the included studies	Yes	Pg 3
Synthesis of results	6	Specify the methods used to present and synthesise results	Yes	Pg 3
**RESULTS**				
Included studies	7	Give the total number of included studies and participants and summarise relevant characteristics of studies	Yes	Pg 3
Synthesis of results	8	Present results for main outcomes, preferably indicating the number of included studies and participants for each. If meta-analysis was done, report the summary estimate and confidence/credible interval. If comparing groups, indicate the direction of the effect (i.e. which group is favoured)	Yes	Pg 3
**DISCUSSION**				
Limitations of evidence	9	Provide a brief summary of the limitations of the evidence included in the review (e.g. study risk of bias, inconsistency and imprecision)	Yes	Pg 3
Interpretation	10	Provide a general interpretation of the results and important implications	Yes	Pg 3
**OTHER**				
Funding	11	Specify the primary source of funding for the review	Yes	Pg 3
Registration	12	Provide the register name and registration number	Yes	Pg 3

## Abbreviations

OA: Osteoarthritis
IA CS: Intra-articular corticosteroids
CS: Corticosteroids
PRP: Platelet rich plasma
HA: Hyaluronic acid
NHS: National Health Service
PRISMA: Preferred Reporting Items for Systematic Reviews and Meta-analyses
WOMAC: Western Ontario and McMaster Universities Osteoarthritis Index
VAS: Visual analogue scale
BMAC: Marrow aspirate concentrate
JBI: Joanna Briggs Institute
ITT: Intention to treat analysis
LA: Local anaesthetic
HIT: Hip Injection Trial
BCT: Best current treatment
KL: Kellgren-Lawrence grade
